# Simulation of Charge Distribution and Microstructure in Semicrystalline Polymeric Ionic-Electronic Conductors Using Classical Simulation at Constant Electrochemical Potential

**DOI:** 10.1021/acs.jctc.5c02111

**Published:** 2026-03-06

**Authors:** Zixuan Wei, Hesam Makki, Paola Carbone, Alessandro Troisi

**Affiliations:** † Department of Chemistry, 4591 University of Liverpool, Crown Street, Liverpool L69 7ZD, UK; ‡ Department of Chemical Engineering, University of Bath, Bath BA2 7AY, UK; § Department of Chemistry, 5292 The University of Manchester, Oxford Road, Manchester M13 9PL, UK

## Abstract

Understanding how charge distributions on aggregated chains change with microstructure under constant electrochemical potential is crucial for elucidating the behavior of polymeric organic mixed ionic–electronic conductors (OMEICs), yet it remains difficult to study. To address this challenge, we introduce a methodology to perform classical atomistic simulations of doped semiconductors at a constant electrochemical potential. The method allows individual polymer chains to be oxidized and reduced, taking into account their individual redox potentials and the externally tunable electrochemical potential. The implementation follows a grand-canonical molecular dynamics (GC-MD) scheme, with the local modulation of the redox potential being described by a QM/MM Hamiltonian. Applied to a semicrystalline polymer with ordered layered and lamellar structures, the method reproduces the experimentally observed minimal structural changes over the electrochemical potentials and charging levels considered. Near the redox potential, charging levels fluctuate more strongly, and variations in the interlamellar angle (defined by the normal of adjacent lamellae) are most pronounced. Moreover, analysis of the local environment reveals no detectable correlation between a chain’s redox reaction and the charge distribution of neighboring chains, except at the most negative potentials, where redox events occur preferentially in more positively charged surroundings. Lastly, examination of individual chains shows minimal chain–chain charge correlation, and the single-chain conformation remains closely linked to its redox behavior. Overall, this work provides a robust framework for investigating charge distributions in dynamically doped systems and offers new conceptual routes for studying polymer structural responses under constant electrochemical potentials.

## Graphical abstract

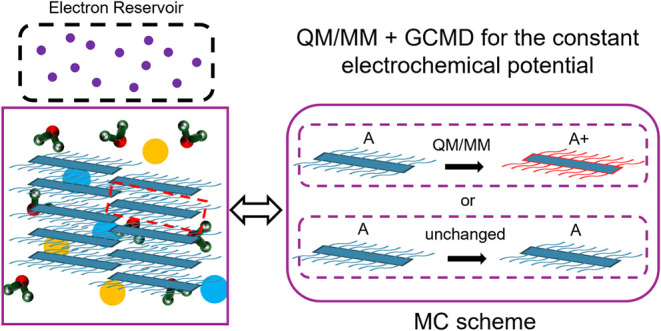

## 1 Introduction

Nowadays, polymeric organic mixed ionic–electronic conductors (OMIECs) have emerged as highly promising candidates for applications in energy storage, electrocatalysis, and biosensing, owing to their unique mixed ionic–electronic conduction mechanism.^1–4^ This unique capability originates from the specific molecular design of such polymers: the conjugated backbone within aggregated chains enables electron exchange with the electrode (the electronic conduction), during which redox reactions occur and dope/dedope charges are released from the chain. The degree of this doping/dedoping is commonly described by the doping level, i.e., the average charge of aggregated chains per repeat unit. Meanwhile, counterions from the electrolyte migrate toward the charged chains to maintain local electroneutrality, constituting the ionic conduction mechanism.^1–9^ The dual-conducting nature of OMIECs facilitates the transduction of ionic signals into electronic currents through volumetric doping processes.^10^

Since the doping/dedoping process alters the charge distribution within chains, the local environment is modified through Coulombic interactions, which in turn affect the polymer structure. The structural stability of the polymer is closely tied to its performance as OMEICs.^3,4^ Consequently, many experimental studies have focused on elucidating the relationships among polymer structure, the local environment, and the doping level.^3,4,11–16^ First, experimental observations^4,15,16^ have shown that the pronounced passive swelling of polymers in OMEICs occurs during the transition from the dry state to immersion in the aqueous electrolyte. After this swelling, the polymer structure undergoes minimal changes and largely maintains its structural stability when the doping level remains low.^3,4^ Cyclic voltammograms (CVs) measurements and repeated doping/dedoping cycles further confirm that this structural stability correlates with stable electronic transport and electrochemical charging behavior in organic electrochemical transistors (OECTs).^3^ However, at high doping levels, strong polaron interactions disrupt this stable structure, leading to an irreversible decrease in conductivity and charge-carrier mobility.^3,16^

The experiments described above provide a macroscopic understanding of how variations in the doping level induce structural changes in the polymer and subsequently influence device performance. Yet, at the microscopic level, even when the overall doping level of the polymer remains relatively constant, individual chains can experience continuous fluctuations in their doping levels due to changes in their structures and local environment. The relationship between such microscopic structural variations and their doping levels remains inaccessible to experimental observation, but it is essential for developing a fundamental understanding of the structure of the OMEICs.

Classical molecular dynamics (MD) simulation is a powerful approach for probing structural variations of polymers on the microscopic scale. Previous MD studies of OMIECs have mainly investigated polymer swelling,^13,14^ side-chain effects,^17,18^ and ion coordination.^19^ However, MD cannot capture the charge distribution within aggregated chains, and obtaining such information typically requires the incorporation of quantum-chemical (QC) methods. Recent progress has led to the development of approaches that combine quantum mechanics (QM) with MD,^20^ which have been successfully applied to inorganic material/aqueous electrolyte interfaces^21–25^ and, more recently, to hydrated OMIECs.^26,27^ Although such hybrid methods are capable of updating the QM-derived charges on aggregated chains at the picosecond time scale in response to the local environment, the doping level of each chain remains a predefined constant (fixed number of electronic charges) throughout the simulation. Therefore, such hybrid methods still fail to realistically simulate the charging process in the case of the OMEICs, where individual chains should hold variable amounts of electronic charge (variable doping levels) due to their continuously evolving structures and local environments. To meet this requirement, an enhanced hybrid framework is needed—one that enables each polymer chain to dynamically redistribute its charge throughout the simulation.

A constant electrochemical potential (Φ) must be incorporated into this enhanced framework because the constant Φ condition naturally drives the electronic charges on individual chains to adjust dynamically in response to their local environments, ultimately yielding a self-consistent charge distribution that reflects the thermodynamic equilibrium expected in real electrochemical settings.^28–30^ In the literature, two main approaches have been developed for performing molecular dynamics simulations under a constant Φ. The first category is the constant Φ method implemented within an MD framework,^31–34^ in which the Φ is maintained by dynamically adjusting the atomic charges on the electrode in response to the position of the electrolyte. Although this approach successfully enforces the mathematical condition of constant Φ and, being entirely MD-based, offers excellent computational efficiency suitable for large-scale simulations, but inherently neglects the quantum-mechanical nature of the electrode.^31^ In contrast, the alternative approach is the Grand Canonical Density Functional Theory (GC-DFT) framework,^30,35–41^ which achieves a constant Φ condition by allowing the electron number in a defined quantum subsystem to fluctuate and by computing the resulting electronic structure self-consistently. In this framework, the system is coupled to an electron reservoir with a constant Φ, enabling electrons to be added into or removed from the quantum region in accordance with the imposed Φ. The method enforces the constant Φ condition and provides a physically rigorous route to modeling electron-transfer processes with full quantum-mechanical fidelity under experimentally relevant electrochemical constraints. While GC-DFT provides a rigorous quantum-mechanical route for simulating electrochemical processes under constant Φ, its computational cost remains prohibitive for large-scale systems.

To balance computational cost and accuracy for a large system of OMEICs under a constant Φ, we introduce such an enhanced framework by integrating the GC scheme of GC-DFT with a QM/MM-embedded constant Φ molecular dynamics approach (QM/MM + GCMD). In this QM/MM + GCMD framework, the quantum subsystem—responsible solely for updating the electronic charge redistribution within chains—is dynamically coupled to an electron reservoir that enforces the constant Φ. Meanwhile, the structural dynamics of OMEICs are simulated through MD. This multiscale, constant Φ framework thus not only enables accurate charge redistribution without explicitly modeling the process of doping—allowing excess charges to spontaneously equilibrate onto the energetically most favorable aggregated chains—but also explores the related structural changes of the polymers.

A recent study^4^ has identified a semicrystalline polymer, poly­[3,3′-bis­(2-(2-(2-methoxyethoxy)­ethoxy)­ethoxy)-2,2′-bithiophene] (P­(g3T2)), whose structural stability contributes significantly to enhanced OMIEC performance, as a new promising model for investigating the relationship between polymer structure and charge distribution in OMEICs. In this study, we construct a model of P­(g3T2) immersed in a NaCl aqueous electrolyte. The redox species are the reduced species of the neutral P­(g3T2)_n_ chain (denoted as species *A*) and the oxidized species of the charged P­(g3T2)_n_ chain (denoted as *A* ^+^). Within our QM/MM + GCMD framework, the relative concentrations of these two species, [A]/[A^+^], are directly regulated by the constant Φ. This setup enables us to probe how microstructural variations—specifically interlayer and interlamellar arrangements—evolve across different doping levels, and to determine whether distinct charge-distribution patterns emerge that correlate with these microstructural changes.

## 2 Methodology

### 2.1 Model Setup

The process of generating and simulating a system comprising ten chains of 10 monomers of g3T2 (P­(g3T2)_10_) immersed in a NaCl solution, as illustrated in Figure 1, involves four main steps. Initially, the molecular structure and corresponding force field for a single P­(g3T2)_10_ were derived using the methodology in ref. 42. The force field details can be obtained from the Supporting Information (Data Availability). Subsequently, five π-stacked backbones of this polymer are constructed with a π–π stacking distance of 0.38 nm^4^. These π–π stacked structures are then organized into two lamellae, arranged with a lamellar spacing of 1.74 nm^4^ consistent with the experimental lamellar spacing in the dry state. Finally, the neutral system is completed by introducing Na^+^and Cl*^–^* ions, and water molecules to maintain a NaCl concentration of 0.1 M.^4^

**Figure 1:**
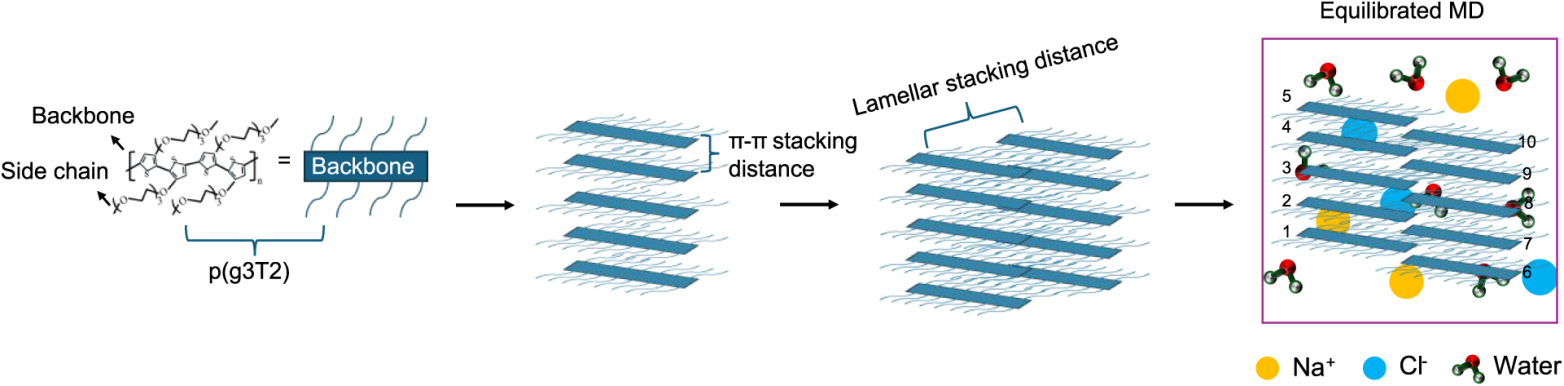
Process for generating hydrated lamellae of P­(g3T2).

### 2.2 QM/MM + GCMD Framework

The system is in contact with an electron reservoir with a constant Φ and the total energy of an electron in the reservoir is −Φe. During the GCMD simulation, one of the two moves interconverting neutral (*A*) and oxidized chain (*A* ^+^) takes place:

1 $$A_{\left(\mathrm{solution}\right)}\to {A^{+}}_{\left(\mathrm{solution}\right)}+{e^{-}}_{\left(\mathrm{reservoir}\right)}\left(\mathrm{oxidation}\right)$$

2 $${A^{+}}_{\left(\mathrm{solution}\right)}+{e^{-}}_{\left(\mathrm{reservoir}\right)}\to A_{\left(\mathrm{solution}\right)}\left(\mathrm{reduction}\right)$$

As in a standard GC approach, once the total energy change, Δ*E*, is defined, for the corresponding move is accepted with probability $\min\left(1,\exp\left(-\frac{\Delta E}{k_{B}T}\right)\right)$ . Note that the addition/removal of an electron does not change the total number of particles, and therefore, there is no term associated with the translational component of the partition function found in Grand-Canonical Monte Carlo (GCMC) or GCMD methods, where atoms/molecules are added or removed.^43–45^

The energy of oxidation of the species *A*,Δ*E*_ox_, can be expressed, if its atomic position is unchanged, as

3 $$\Delta E_{\mathrm{ox}}=-E\left(A\right)+E\left(A^{+}\right)-\Phi e=-E\left(A\right)+E\left(A^{+}\right)-\Phi^{0}e-\Delta \Phi e$$

where Φ^0^ is a redox potential (when Φ = Φ^0^ the concentration of oxidized and reduced species is equal), and *E*(*A*) and *E*(*A* ^+^) are the total energies of the neutral and oxidized species. Correspondingly, the instantaneous reduction energy is

4 $$\Delta E_{\mathrm{red}}=E\left(A\right)-E\left(A^{+}\right)+\Phi^{0}e+\Delta \Phi e$$

When the electrochemical potential is equal to the redox potential (ΔΦ = 0), the average oxidation and reduction energies are the same, i.e.

5 $$\langle -E\left(A\right)+E{(A^{+})\rangle }_{A}-\Phi^{0}e=\langle E\left(A\right)-E{(A^{+})\rangle }_{A^{+}}+\Phi^{0}e$$

Note that for the oxidation, the average runs over all the neutral species, ⟨⟩_*A*_, and for the reduction, over the cationic species, ⟨⟩_*A*_ This condition enables the definition of the equilibrium potential as

6 $$\Phi^{0}e=\frac{1}{2}\left(\langle -E\left(A\right)+E{(A^{+})\rangle }_{A}-\langle E\left(A\right)-E{\left(A^{+})\right\rangle }_{A^{+}}\right)$$

For the implementation in the context of large-scale atomistic MD simulation, the energy difference *E*(*A* ^+^) – *E*(*A*) can be approximated as the HOMO energy of the species *A*, a quantity that can be computed in the presence of the surrounding point charges in a QM/MM scheme to include both the effect of the environment and that of the intramolecular concentration. The quality of this approximation for polymeric chains was checked in Figure S1 and also confirmed from the ref. 26. The removal of an electronic charge changes the electrostatic interaction with the surrounding chains via a different distribution of point charges but also, in principle, modifies the intramolecular potential. The latter term can be associated with the intrachain polaron stabilization energy. In the present framework, this effect is treated by assuming that the intrachain polaron stabilization energy constitutes an approximately constant offset for chemically identical polymer chains. Under this assumption, the stabilization energy can be included into the reference (zero) of the electrochemical potential and therefore does not affect the relative redox probabilities between different chains at a given applied potential ΔΦ. This approximation is justified for the systems considered here, where all polymer chains share the same chemical structure and where local conformational fluctuations are sampled dynamically during the MD simulations. Importantly, as shown in Figure 5, the conformational differences between chains remain small within the low doping levels investigated in this work. Furthermore, polaronic effects are also limited by the charge delocalization at low doping for these relatively long chains: the average charge per thiophene is 0.025*e* if the chain is oxidized, and the largest charge rarely exceeds 0.1*e*. This approximation would become inadequate if the polaron stabilization energy varied strongly between chains, for example, due to large conformational heterogeneity or strong, localized environmental effects. In such cases, explicit treatment of intrachain structural relaxation upon redox events would be required. These regimes refer to high doping levels; however, they are beyond the scope of the present study.

The process of computing the value of Φ^0^ *e* is shown in Figure 2.

**Figure 2:**
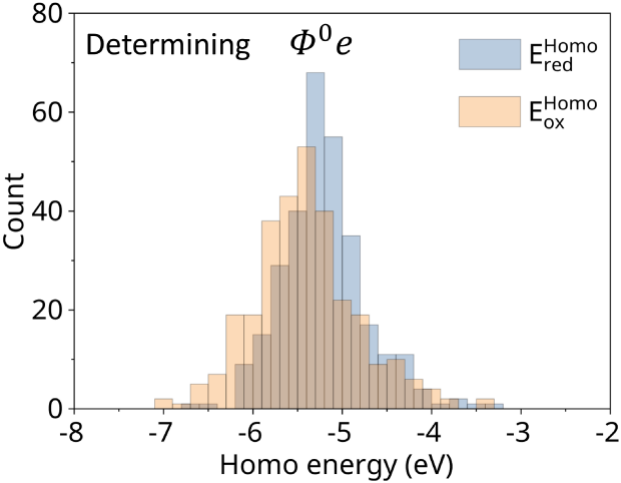
Determination of Φ^0^ *e* (redox potential energy) follows eq 6, where $E_{\mathrm{red}}^{\mathrm{HOMO}}$ and $E_{\mathrm{ox}}^{\mathrm{HOMO}}$ denote the HOMO energies entering the terms ${\left(E\left(A\right)-E\left(A^{+}\right)\right)}_{A^{+}}$ and (−*E*(*A*) + *E*(*A* ^+^))_*A*_, respectively. Their time-averaged values correspond to $\langle E\left(A\right)-E\left(A^{+}\right)\rangle_{A^{+}}$ and ⟨−*E*(*A*) + *E*(*A* ^+^)⟩_*A*_.

The overall QM/MM + GCMD framework consists of seven steps, as shown in Figure 3:

(i) The system evolves with a classical MD for a time interval Δ*t*.
(ii) A chain *i* that undergoes a redox reaction is selected randomly (if oxidized, the reduction is attempted; if reduced, the oxidation is attempted).
(iii) The energy change for the redox reaction (Δ*E*_ox_/Δ*E*_red_ if chain *i* is reduced/oxidized) is computed according to eq 3/eq 4. Specifically, the HOMO energy in Δ*E*_ox_/Δ*E*_red_ is calculated using the QM/MM scheme.^27^
(iv) The move is accepted with the probability $P=\min\left(1,\exp\left(-\frac{\Delta E_{ox}or\Delta E_{red}}{k_{B}T}\right)\right)$ .
(v) If the move is accepted, the force field of the system is modified as follows: the charge on chain *i* increases by 1*e* in case of oxidation (QM/MM scheme) or decreased by −1*e* in case of reduction. The total charge of the system is adjusted accordingly by ± 1*e*. This excess charge is uniformly distributed over all oxygen atoms of the water molecules to neutralize the system. We adopt this mean-field charge neutralization because it prevents the requirement to insert or delete a particle (a process requiring a separate optimization and that generally reduces the probability of acceptance of the move).^43–45^ Considering the number of water molecules in the simulation box, this means ± 0.000027 *e* on each oxygen atom. This small change in the original water force field does not lead to any significant change in the water properties or the local ion-polymer structure (as shown in Figure S2 with the detailed discussion).
(vi) If the move is rejected, the force field of the system remains unchanged.
(vii) The process is repeated from (i) for a desired total simulation time.

**Figure 3:**
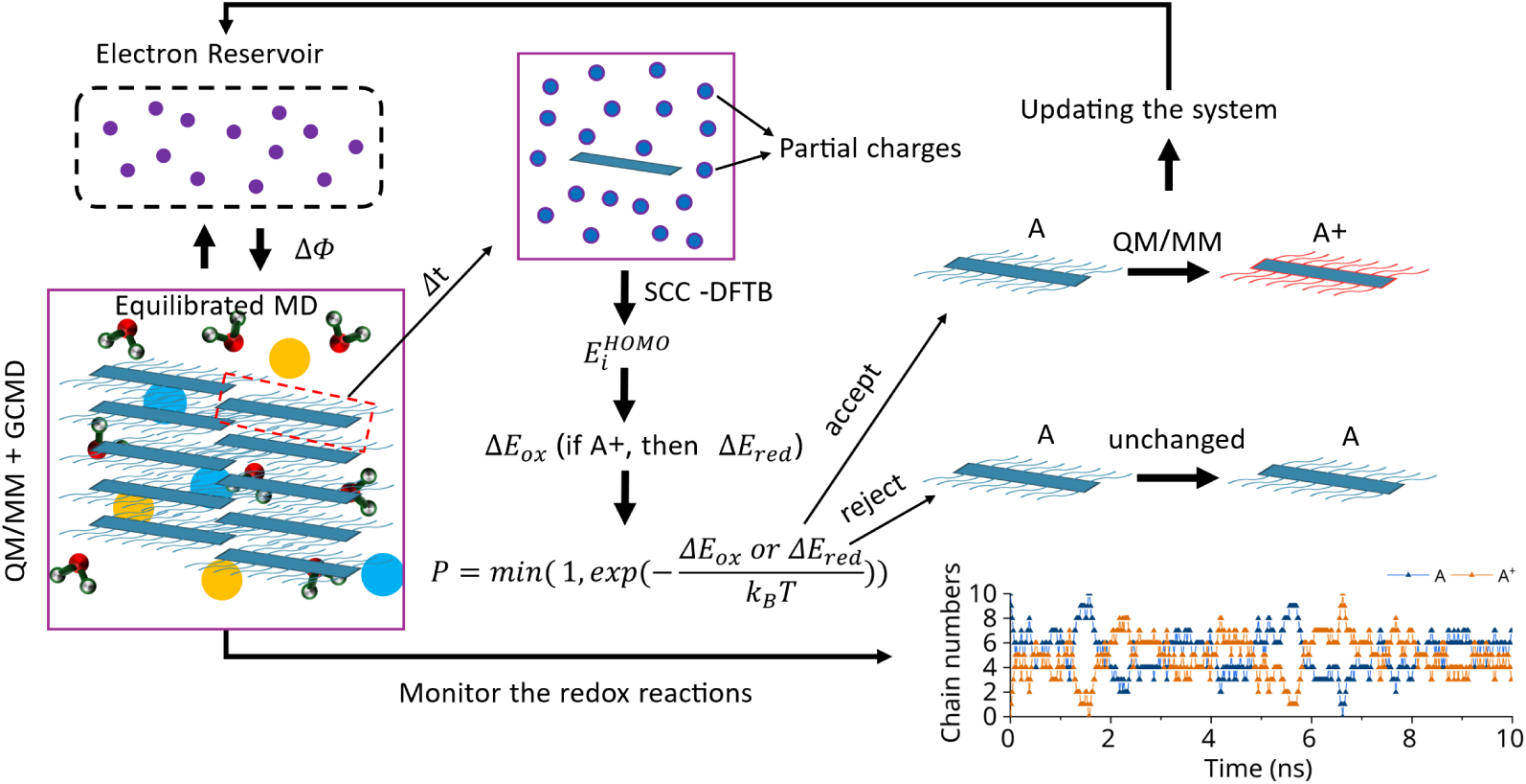
Process for QM/MM + GCMD algorithm following step (i)–(vii).

This algorithm precisely reproduces the effect of Fermi level controlling which chains are preferentially targeted. While trial redox moves are proposed by randomly selecting chains, the acceptance probability is strictly dictated by the local redox energy relative to the Fermi level (i.e., the electrostatic potential). As a result, chains whose instantaneous redox energies lie closer to the Fermi level are preferentially oxidized or reduced, whereas energetically unfavorable chains are systematically rejected.

For the implementation of this framework, one also needs to define an approach to change the atomic charges in the force field when the total charge on a species is changed in step (v). Here, following the procedure of ref.^27^.

7 $$q_{1,i}^{FF}\left(t\right)=q_{0,i}^{FF}+\left(q_{1,i}^{M}\left(t\right)-q_{0,i}^{M}\right)$$

The $q_{1,i}^{FF}\left(t\right)$ denotes the force-field point charges of the atoms in chain *i* when the chain carries a + 1*e* charge at time *t*, while $q_{0,i}^{FF}$ represents the time-independent force-field point charges of the atoms in the neutral chain *i*. The $q_{1,i}^{M}\left(t\right)$ is the Mulliken charge distribution for the +1*e* charged chain *i*, computed using a QM/MM scheme that accounts for the local environment (i.e., the surrounding point charges) at time *t*. Similarly, $q_{0,i}^{M}$ is the Mulliken charge distribution for the chain *i* in its reference equilibrium geometry for the isolated neutral chain, which is time independent. For the increased −1*e* of the reduction, the charge of the chain *i* returns to the $q_{0,i}^{FF}$ .

Finally, for the specific QM/MM scheme, two parts are included. First, in step (iii), the HOMO energy of the selected chain is computed while explicitly accounting for its local environment, represented by the surrounding point charges. Second, in step (v), when the chain undergoes oxidation, the excess Mulliken charge $\left(q_{1,i}^{M}\left(t\right)-q_{0,i}^{M}\right)$ associated with the oxidized state is computed in the same embedded QM/MM environment (including surrounding point charges). The choice of the QC method in this QM/MM scheme should be consistent with performing a large number of evaluations of the HOMO energy and computing excess Mulliken charge over the computational cost of the simulation. We therefore adopt here the SCC-DFTB as an efficient approximation of the Kohn–Sham density functional theory (DFT),^46^ commonly used in applications of this nature.^46,47^

It should be noted that eq 6 allows the determination of the electrostatic potential where the concentration of reduced and oxidized species is the same within all of the approximations. In this way, it is possible to use the simulation to evaluate which chains are preferentially oxidized/reduced, and the potential is displaced from the average redox potential by ΔΦ The approach will capture the effect of different chains having different “instantaneous” redox potentials due to their conformation or electrostatic interaction with the surroundings, going beyond the current approach of randomly distributing excess charge on doped polymers.^16,48,49^

Within this framework, acceptance of a Monte Carlo redox move results in an instantaneous update of force-field charges and, consequently, discontinuous changes in the electrostatic forces between successive MD segments. Such force discontinuities are intrinsic to GC-MD schemes.^43–45^ Importantly, these force jumps do not compromise ensemble sampling, as demonstrated by the temperature stability tests (Figure S3) discussed in Section S3 of the Supporting Information.

### 2.3 Further Computational Details

For the equilibrated MD simulation of hydrated OMEICs in Section 2.1, the box size is 20 × 8 × 5 nm. The periodic boundary condition is applied in all directions. The equilibration NPT simulation is 10 ns long with a time step of 2 fs after a 5 ns NVT simulation under the MD simulation (GROMACS, version 2022).^50^ The Nosé–Hoover thermostat^51^ is employed to fix the temperature at 298.15 K with relaxation times of 0.5 ps. The Parrinello–Rahman barostat^52^ is employed to fix the isotropic pressure at 1 bar and compressibility of 4.5 × 10^–5^ bar^–1^. The Verlet cutoff scheme^53^ with the cutoff distance of short-range Lennard–Jones and electrostatic interactions is set to 1.2 nm. The long-range electrostatic interaction is treated by the Particle Mesh Ewald approach (PME).^54^ The switch function of the Lennard–Jones 12–6 potential starts at 1 nm and smoothly truncates at 1.2 nm. The switch function of the electrostatic potential is applied at 1.2 nm. The SPC/E^55^ is used as the water model with the rigid constraint by the SETTLE algorithm, and the force field of ions comes from the OPLS.^56^ For determining Φ^0^ *e* in Section 2.2, an MD (50% oxidized chains in the system) is run for 10 ns on the equilibrated configuration under the NPT ensemble. All setups of parameters are the same as those in the equilibrated MD simulation.

The QM/MM + GCMD framework in Section 2.2 runs on the equilibrated configuration, where all chains are reduced. In each loop of this framework, the QM/MM scheme at the SCC-DFTB level in this algorithm employed the DFTB+ software package.^57^ The convergence threshold of the self-consistent charge optimization is set to 1 × 10^–5^ Hartree. The following GCMD runs 20 ps (Δ*t* = 20 ps) under the NPT ensemble, and all setups of parameters are the same as the MD simulation above. To investigate the structural change and charge distribution in varied doping levels, seven independent simulations (QM/MM + GCMD) were performed at ΔΦ values of −0.06, −0.02, −0.01, 0.00, 0.01, 0.02, and 0.06 V. The desired total simulation time was 10 ns (500 loops), and the last 9 ns (450 loops) was used for analysis.

## 3 Results

### 3.1 Initial Testing of the QM/MM + GCMD Framework

Before performing more detailed analyses of the polymeric microstructures with their charge distributions, it is essential to verify that the charge distribution obtained from QM/MM and GCMD simulations is consistent with theoretical expectations. Under a constant Φ condition, the relative concentrations of the neutral and oxidized species should be governed by electrochemical equilibrium. Therefore, we compute the average concentration ratio between neutral and oxidized chains, <[*A*]>/<[*A* ^+^]> obtained after equilibration in the QM/MM + GCMD framework. This ratio is then compared with the [*A*]/[*A* ^+^] ratio predicted by the Nernst equation (*NE*) in eq 8, which provides the fundamental theoretical relationship between redox species at a given electrochemical potential.

8 $$\Phi =-\frac{RT}{F}\ln\left(\frac{\left[A\right]}{\left[A^{+}\right]}\right)$$

where *R* and *F* are the universal gas constant and the Faraday constant.

Agreement between the two as a function of ΔΦ (as shown in Figure 4a) demonstrates that the QM/MM + GCMD framework correctly reproduces the equilibrium proportion of oxidized and neutral species. At the most negative and positive ΔΦ (−0.06 and 0.06 V), reduction and oxidation predominantly govern the redox process, reflected by the largest and smallest values of <[*A*]>/<[*A* ^+^]>, respectively. When ΔΦ is zero, <[*A*]>/<[*A* ^+^]>is around 1, which correctly indicates that the oxidation and reduction have similar rates.

**Figure 4:**
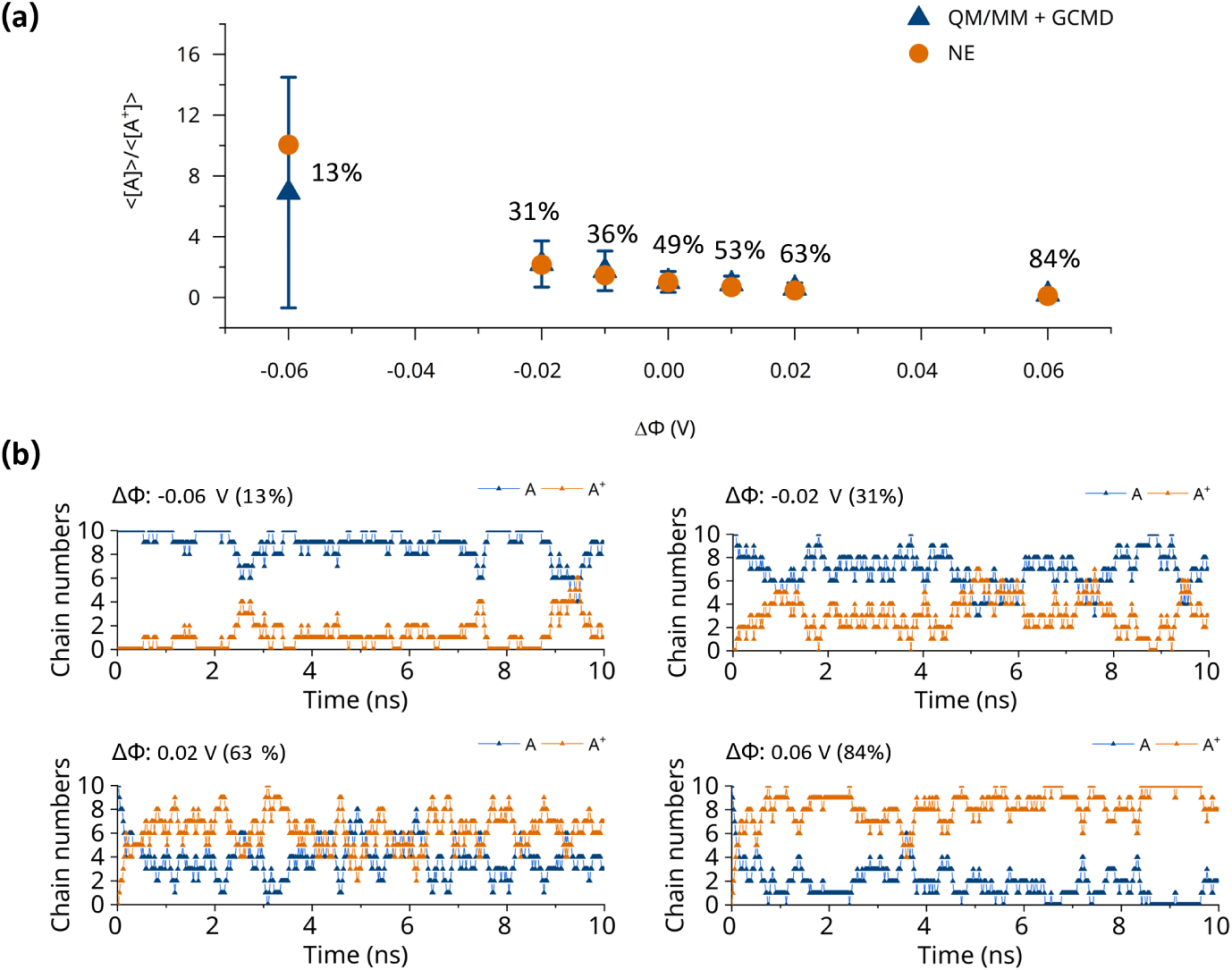
(a) The <[*A*]>/<[*A* ^+^]>that was simulated from the QM/MM + GCMD framework and predicted from the *NE* as the function of ΔΦ. These related oxidation levels are also shown. (b) The varied chain numbers under different ΔΦ as the function of time.

Figure 4b for four ΔΦ values and Figure S4 for the other tested values, provide detailed insights into the redox dynamics within the system, allowing us to directly observe the full redox process. Throughout the simulations, the total number of *A* and *A* ^+^ varies in response to different ΔΦ. When |ΔΦ| is relatively large, the number of *A* and *A* ^+^ slightly fluctuates with a lower frequency of redox reactions. In contrast, when |ΔΦ| is close to zero, the fluctuations in the numbers of *A* and *A* ^+^ are noticeably larger with a higher frequency of redox reactions. In summary, the QM/MM + GCMD framework accurately captures the full redox dynamics and reproduces the equilibrium charge distribution in accordance with *NE*.

### 3.2 Polymer Microstructure at Different ΔΦ

Understanding how polymer microstructures respond to changes in electrochemical potentials is essential for elucidating OMIEC-based devices.^3,4^ We therefore first explore the change in the polymeric microstructure in the range of ΔΦ between −0.06 and 0.06 V, where the oxidation level varies between 13% and 84% (on average). We also consider the extreme cases in which all chains remain either fully reduced (0% oxidation level) or fully oxidized (100% oxidation level) throughout the MD simulation. Each production NPT simulation was run for 10 ns, and the last 9 ns was used for analysis (the charge of oxidized chains using $q_{1,i}^{\mathrm{FF}}\left(t\right)$ ). In the following analysis, the oxidation level is used to represent the value of ΔΦ.

To quantify how the polymer’s microstructure changes both within layers (π*-*π stacking) and between lamellae, we compute two sets of structural descriptors (illustrated in Figure 5a,c).

**Figure 5:**
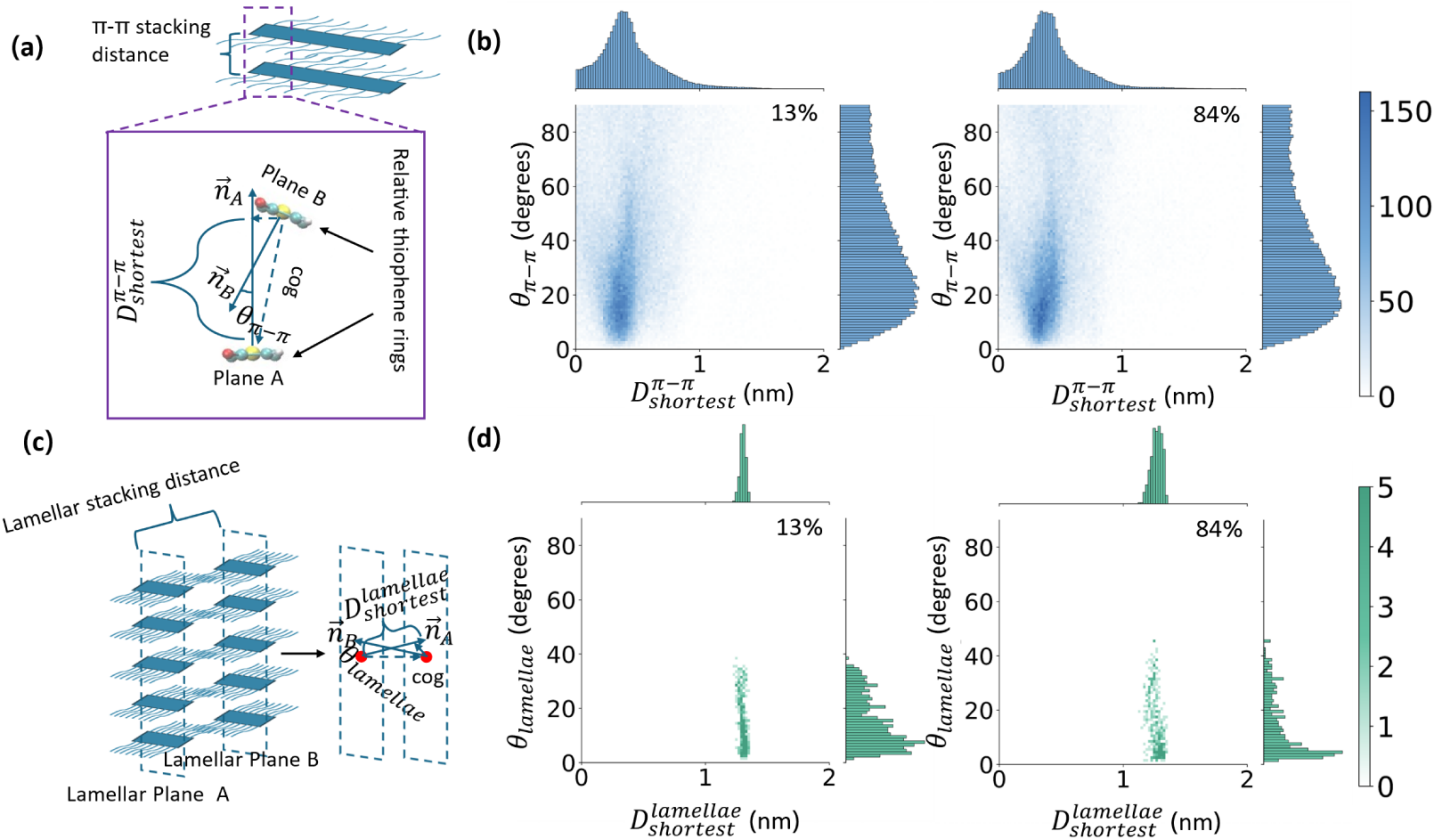
(a) Illustration of calculating $D_{\mathrm{shortest}}^{\pi -\pi }$ and θ_π*‑*π_. (b) The distribution of θ_π*‑*π_ with $D_{\mathrm{shortest}}^{\pi -\pi }$ in thiophene rings under different oxidation levels of 13% and 84% (heatmap), while the top plot of each figure is the distribution of $D_{\mathrm{shortest}}^{\pi -\pi }$ and the right plot of each figure is the distribution of θ_π*‑*π_. (c) The illustration of calculating $D_{\mathrm{shortest}}^{\mathrm{lamellae}}$ and θ_lamellae_. (d) The distribution of θ_lamellae_ with the $D_{\mathrm{shortest}}^{\mathrm{lamellae}}$ between lamellae under different oxidation levels of 13% and 84% (heatmap), while the top plot of each figure is the distribution of $D_{\mathrm{shortest}}^{\mathrm{lamellae}}$ and the right plot of each figure is the distribution of θ_lamellae_.

Interlayer descriptors:

1. Interlayer angle (θ_π*‑*π_): the angle between the normal vectors of two thiophene ring planes;
2. Shortest interlayer distance $\left(D_{\mathrm{shortest}}^{\pi -\pi }\right)$ : the distance obtained by projecting the center of geometry (cog) of one thiophene ring onto the normal vector of the other.

These metrics are calculated for all thiophene ring pairs across different chains (40 rings per chain) to capture changes in π-π stacking.

Interlamellar descriptors:

1. Interlamellar angle (θ_lamellae_): the angle between the normal vectors of two lamellae;
2. Shortest interlamellar distance $\left(D_{\mathrm{shortest}}^{\mathrm{lamellae}}\right)$ : defined similarly through cog projection onto the lamellar normal.

We do not observe significant differences in the distribution of the four parameters above, as shown in Figure 5b,d for two oxidation levels (13% and 84%) and in the Supporting Information in Figures S5, S6 for others. The θ_lamellae_ comparably presents a largest fluctuation among the four parameters. Intuitively, the θ_lamellae_ would be expected to become increasingly disordered with higher oxidation levels due to enhanced Coulombic repulsion between lamellae. However, the time-averaged interlamellar angle, θ̅_lamellae_, exhibits the larger value (larger disorder) within the intermediate oxidation level between 36% and 53% (in Figure 6) due to the larger fluctuation of the charging level at this range (as shown in Figure S4).

**Figure 6:**
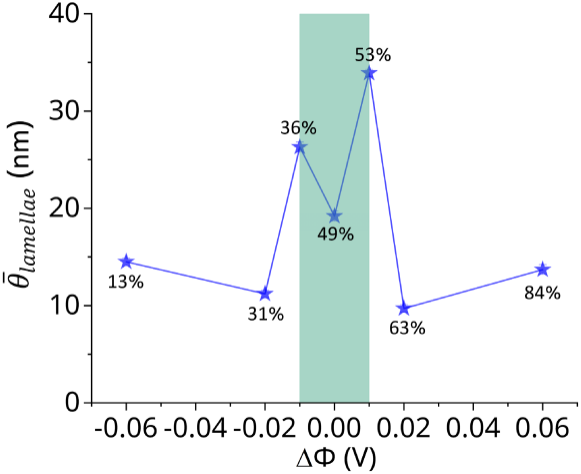
θ̅_lamellae_ as the function of the oxidation level (ΔΦ). The light green region represents the more disordered region.

Two additional descriptors that are readily correlated with the experimental observations^4^ are time-averaged π-π stacking (*D̅*_π‑π_) and lamellar stacking distances. These two quantities (Figure 7a,c) are obtained by time-averaging all π-π stacking and lamellar stacking distances that satisfy the criteria defined in our previous work.^42^ The average π-π stacking distance (*D̅*_π‑π_) is calculated for all those cases for which the θ_π*‑*π_ is less than 10° or greater than 170° (parallel planes), $D_{\mathrm{shortest}}^{\pi -\pi }$ is smaller than 0.5 nm, and the horizontal distance $\left(H_{\mathrm{cog}}^{AB}\right)$ between the cog of each parallel pair of thiophene rings is less than 0.5 nm.

**Figure 7:**
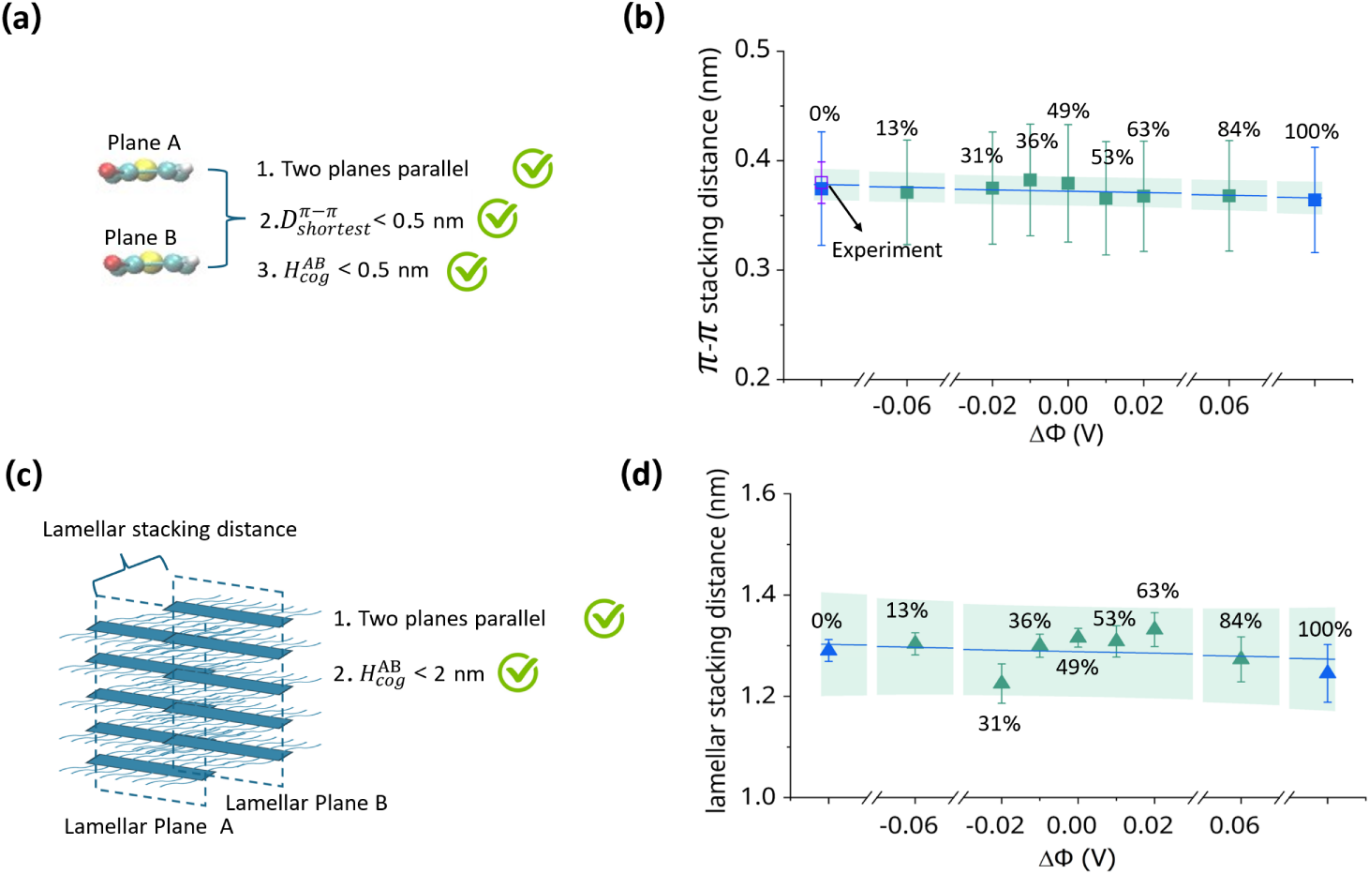
(a) The illustration of the criteria for the calculation of the averaged π*-*π stacking distance, *D̅*_π‑π_. (b) The time-averaged π*-*π stacking distance as the function of oxidation level; the blue-filled square represents the case using MD, and the green-filled square represents the case using QM/MM + GCMD framework. The blue line is the linear fit across all data points with a green prediction band. (c) The illustration of counting the time-averaged lamellar stacking distance with the criteria. (d) The time-averaged lamellar stacking distance as a function of oxidation level; the blue-filled triangle represents the case using MD, and the green-filled triangle represents the case using QM/MM + GCMD framework. The blue line is the linear fit across all data points with a green prediction band.

Average lamellar stacking distance is calculated when θ_lamellae_ is less than 30° or greater than 150° (parallel planes), and the horizontal distance $\left(H_{\mathrm{cog}}^{AB}\right)$ between the cog of two lamellar planes is smaller than 2 nm.

In Figure 7b, the *D̅*_π‑π_ of the fully reduced case agrees with the experimental value^4^ for the same polymer in 0.1 M NaCl solution. Moreover, *D̅*_π‑π_ remains essentially constant from the fully reduced state (0.37 ± 0.05 nm) to the fully oxidized state at a low carrier density of 0.025 charges per thiophene ring (0.36 ± 0.05 nm). Overall, *D̅*_π‑π_ fluctuates around 0.4 nm, indicating minimal changes in the interlayer structure, consistent with experimental observations^4^ and previous MD simulations^16^ on hydrated P­(g3T2), which also reports ∼0.4 nm across a wide range of low carrier densities.

Similarly, Figure 7d shows that the average lamellar stacking distance remains nearly unchanged, varying only from 1.29 ± 0.02 nm (fully reduced) to 1.25 ± 0.06 nm (fully oxidized) and consistently near 1.3 nm. This small variation suggests negligible interlamellar structural changes, in agreement with the previous experiment on p­(g2T-TT)^3^ and recent studies on P­(g3T2)^4^ at low carrier densities. Although both simulations and experiments show minimal structural changes, the absolute lamellar stacking distance differs: experiments report ∼3 nm due to water and ion uptake that induces swelling,^4^ whereas our simulations do not capture this effect. This discrepancy is likely due to the nanosecond simulation time scale, which is insufficient to model the much slower water/ion diffusion and consequent stack rearrangement observed experimentally. This limitation is also reported in previous MD studies^16^ at low carrier densities that found lamellar distances between 0.8 and 1.4 nm—consistent with the ∼1.3 nm value reported here.

Overall, our simulations reproduce the minimal structural changes upon oxidation and accurately capture the absolute π-π stacking distance in agreement with previous experimental and simulation studies.

### 3.3 Understanding the Charge Distribution for Different ΔΦ

After characterizing the polymer microstructure, we wished to study whether oxidation or reduction preferentially occurs adjacent to chains that are oxidized or reduced. For every oxidation or reduction step, we evaluated the average charge of the nearest neighboring chains along the π-π stacking and the lamellar stacking directions, as illustrated in Figure 8a–c.

**Figure 8:**
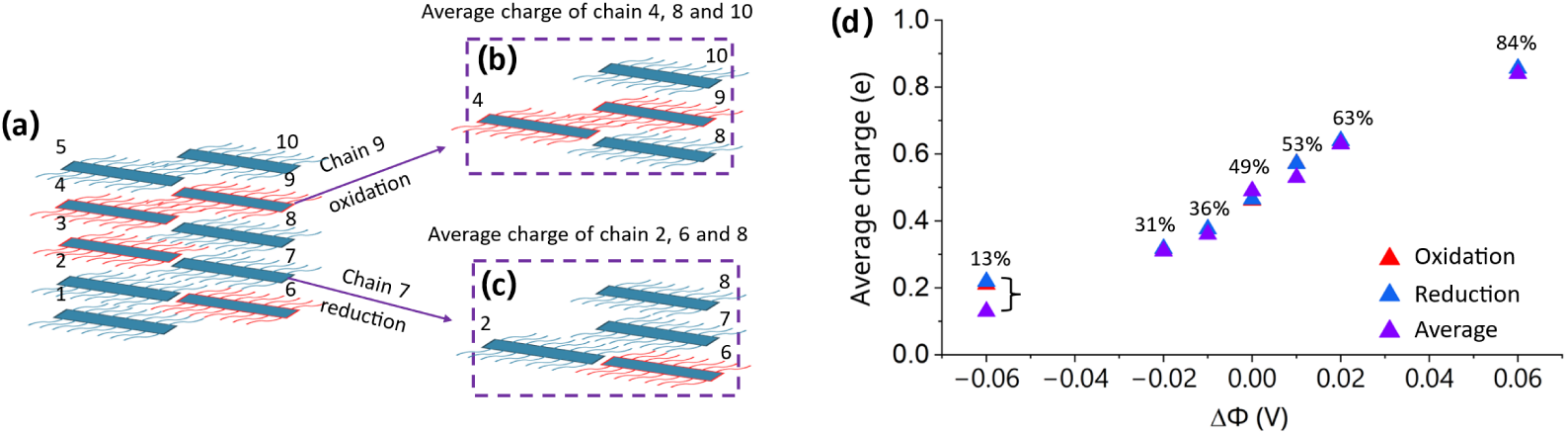
(a–c) The illustration of calculating the average charge of the nearest neighboring chains (both π*-*π stacking and the lamellar stacking directions) at the oxidation or reduction step. (d) The time-averaged charge of neighboring chains for oxidation and reduction, with the overall time-averaged chain charge as the function of ΔΦ (the corresponding oxidation level is also marked in the figure).

For each ΔΦ, we then separately compute the time-averaged neighboring charge for all chains undergoing oxidation and reduction, and compare these values with the overall time-averaged chain charge (Figure 8d). For nearly all ΔΦ, the average neighboring charges associated with oxidation and reduction (red and blue triangles) closely match the overall average (purple triangles). This indicates that redox events do not preferentially occur next to chains with any particular charge state. Only at the most negative ΔΦ (−0.06 V), corresponding to the strongest reducing conditions, we observe a discrepancy: redox events are associated with a more positively charged local environment.

Furthermore, it is valuable to examine how charge distributions within individual chains evolve during redox processes under varying ΔΦ. As shown in the top row of Figure 9 for three oxidation levels and Figure S7 for others, the probability of each chain being oxidized (i.e., the fraction of time spent in the oxidized state) increases with ΔΦ. This trend is visually represented by the increasingly dark blue regions progressing from left to right. When |ΔΦ| is relatively large or close to zero, each chain exhibits correspondingly lower or higher frequencies of redox reactions, consistent with the findings identified in Figure 4b based on the total number of changes in redox chains.

**Figure 9:**
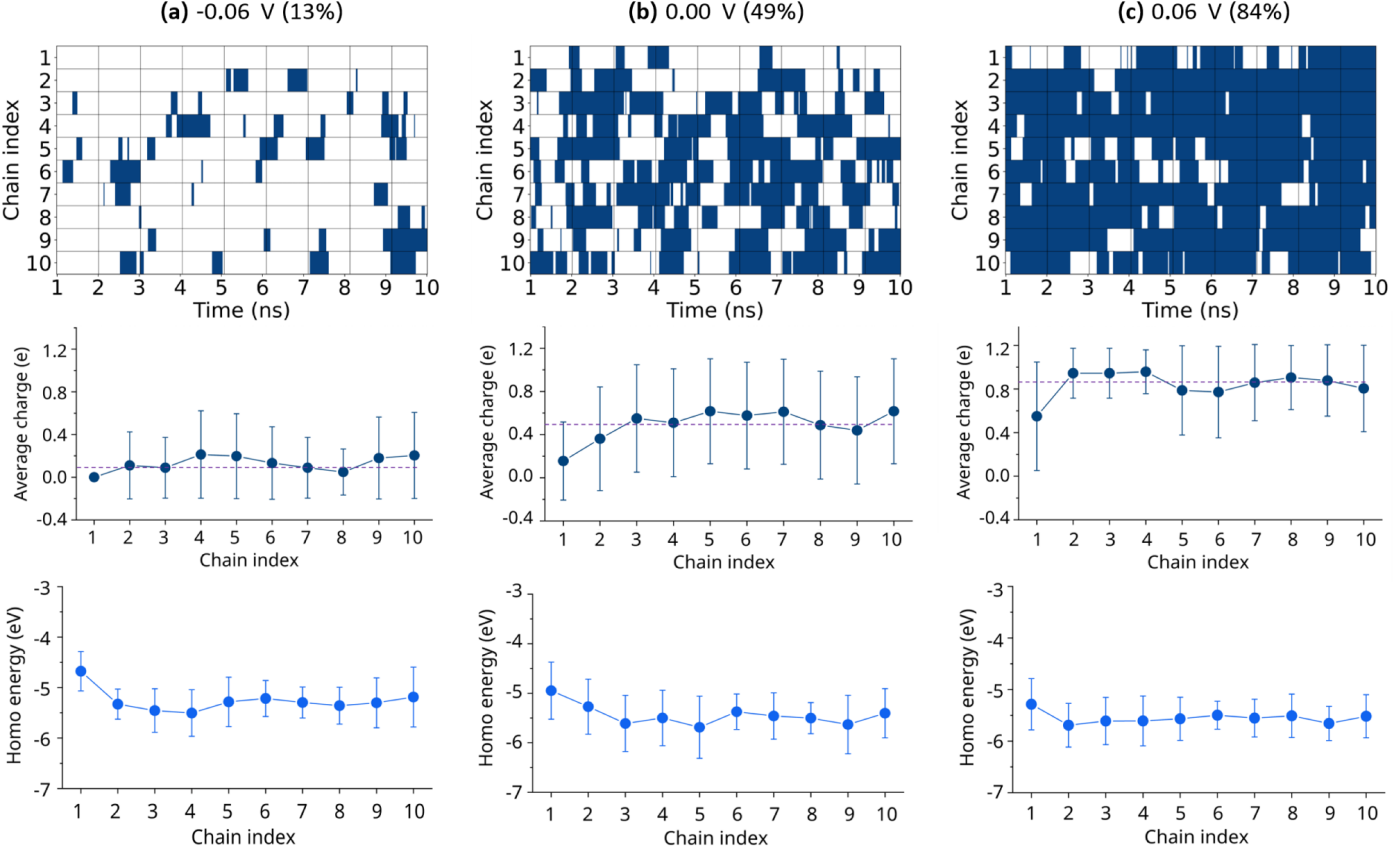
(a–c) The data of the three figures in each column correspond to the same ΔΦ value reported at the top of each column. The first row of figures shows the charge distribution of each chain as a function of time, where the dark blue and white represent the oxidized and reduced states, respectively. The second row of figures represents the time-averaged charge of each chain as a function of ΔΦ with the standard deviation. The dashed purple line represents the overall average charge value in all chains. The third row of figures represents the time-averaged HOMO energy of each chain undergoing redox reactions as a function of ΔΦ with the standard deviation.

To quantify variations in oxidation probability among chains at each ΔΦ, the time-averaged charge of each chain is computed (Figure 9 and Figure S7, second row). Across all ΔΦ, this value fluctuates around the dashed purple line representing the overall time-averaged chain charge, confirming the presence of chain-to-chain variations. One chain, characterized by a larger structural disorder, consistently exhibits a lower average charge. Notably, its neighboring chains do not show compensatory increases; their average charges remain close to the overall mean. This indicates that local structural disorder in a single chain does not significantly influence the redox behavior of adjacent chains and that interchain structural correlations in charge distribution are minimal.

To further investigate energy-related oxidation probability, we compute the time-averaged HOMO energy of each chain during oxidation and reduction at different ΔΦ (Figure 9 and Figure S7, third row). Most chains exhibit HOMO energies narrowly distributed around −5.5 eV, largely insensitive to the oxidation probability. In contrast, the more disordered chain shows a distinctly lower HOMO energy, demonstrating that structural disorder modifies the HOMO energetics. Such a finding is consistent with previous observations,^26^ suggesting that certain conformations render specific chains persistently more susceptible to oxidation or reduction than others.

## 4 Conclusions

In conclusion, we developed a QM/MM + GCMD framework that enables direct simulation of dynamic charging in polymer chains under constant ΔΦ and distributes charge according to thermodynamic preference. Applied to semicrystalline polymers, the framework reproduces the experimentally observed minimal structural response over the ΔΦ: both interlayer and interlamellar stacking metrics remain largely stable, while the interlamellar angle is the most responsive descriptor and becomes most disordered at the ΔΦ close to zero. Moreover, our local environment analysis shows no detectable correlation between a chain’s redox event and the charge state of its nearest neighboring chains, except at the most negative ΔΦ where redox events occur preferentially in more positively charged surroundings. At the single-chain level, we also observe minimal chain–chain charge correlation, whereas the conformation of an individual chain remains closely linked to its redox behavior. Finally, while the framework accurately captures charge distributions, its structural predictions remain constrained by the nanosecond time scales accessible to MD: the slow morphological processes such as lamellar swelling or delamination are not accessible. Overall, QM/MM + GCMD provides a robust platform to investigate the correlation between charge distributions and microstructural changes in dynamically doped polymers under constant electrochemical potential.

## Supplementary Material





## Data Availability

The complete force field files for P­(g3T2) in the system, including the GROMACS.gro,.itp, and.top files, are available in the Supporting Information. These files contain all functional forms and parameter values required to reproduce the simulations reported in this work.

## Notes

Detailed information on the validation tests of approximation of using HOMO energy, neutralized approach, temperature, varied chain numbers under different ΔΦ as the function of time, the distribution of the θ_π*‑*π_ with the $D_{\mathrm{shortest}}^{\pi -\pi }$ in thiophene rings under different oxidation levels, the distribution of the θ_lamellae_ with the $D_{\mathrm{shortest}}^{\mathrm{lamellae}}$ between lamellae under different oxidation levels, the charge distribution of each chain as a function of time, the time-averaged charge of each chain as a function of the ΔΦ, the time-averaged HOMO energy of each chain as a function of the ΔΦ (PDF)
Forcefiels of P­(g3T2) (ZIP)

## References

[1] Paulsen B. D., Tybrandt K., Stavrinidou E., Rivnay J. (2020). Organic Mixed Ionic–Electronic
Conductors. Nat. Mater..

[2] Tropp J., Meli D., Rivnay J. (2023). Organic Mixed
Conductors for Electrochemical
Transistors. Matter.

[3] Quill T. J., LeCroy G., Marks A., Hesse S. A., Thiburce Q., McCulloch I., Tassone C. J., Takacs C. J., Giovannitti A., Salleo A. (2024). Charge Carrier Induced Structural Ordering and Disordering
in Organic Mixed Ionic Electronic Conductors. Adv. Mater..

[4] Tsarfati Y., Bustillo K. C., Savitzky B. H., Balhorn L., Quill T. J., Marks A., Donohue J., Zeltmann S. E., Takacs C. J., Giovannitti A., McCulloch I., Ophus C., Minor A., Salleo A. (2025). The Hierarchical
Structure of Organic Mixed Ionic–Electronic
Conductors and Its Evolution in Water. Nat.
Mater..

[5] Moia D., Giovannitti A., Szumska A. A., Maria I. P., Rezasoltani E., Sachs M., Schnurr M., Barnes P. R. F., McCulloch I., Nelson J. (2019). Design and Evaluation of Conjugated
Polymers with Polar
Side Chains as Electrode Materials for Electrochemical Energy Storage
in Aqueous Electrolytes. Energy Environ. Sci..

[6] Balhorn L., MacPherson Q., Bustillo K. C., Takacs C. J., Spakowitz A. J., Salleo A. (2022). Closing the Loop between Microstructure and Charge
Transport in Conjugated Polymers by Combining Microscopy and Simulation. Proc. Natl. Acad. Sci. U. S. A..

[7] Kim S.-M., Kim C.-H., Kim Y., Kim N., Lee W.-J., Lee E.-H., Kim D., Park S., Lee K., Rivnay J. (2018). Influence of PEDOT: PSS Crystallinity and Composition
on Electrochemical Transistor Performance and Long-Term Stability. Nat. Commun..

[8] Zhu M., Li P., Li J.-L., Lei T. (2022). Molecular Packing and Film Morphology
Control in Organic Electrochemical Transistors. Mol. Syst. Des. Eng..

[9] Nielsen C. B., Giovannitti A., Sbircea D.-T., Bandiello E., Niazi M. R., Hanifi D. A., Sessolo M., Amassian A., Malliaras G. G., Rivnay J., McCulloch I. (2016). Molecular
Design of Semiconducting Polymers for High-Performance Organic Electrochemical
Transistors. J. Am. Chem. Soc..

[10] Cea C., Spyropoulos G. D., Jastrzebska-Perfect P., Ferrero J. J., Gelinas J. N., Khodagholy D. (2020). Enhancement-Mode
Ion-Based Transistor as a Comprehensive
Interface and Real-Time Processing Unit for in Vivo Electrophysiology. Nat. Mater..

[11] Quill T. J., LeCroy G., Halat D. M., Sheelamanthula R., Marks A., Grundy L. S., McCulloch I., Reimer J. A., Balsara N. P., Giovannitti A., Salleo A., Takacs C. J. (2023). An Ordered, Self-Assembled Nanocomposite
with Efficient Electronic and Ionic Transport. Nat. Mater..

[12] Paulsen B. D., Giovannitti A., Wu R., Strzalka J., Zhang Q., Rivnay J., Takacs C. J. (2021). Electrochemistry
of Thin Films with
In Situ/Operando Grazing Incidence X-Ray Scattering: Bypassing Electrolyte
Scattering for High Fidelity Time Resolved Studies. Small.

[13] Ghosh S., Zozoulenko I. (2020). Effect of
Substrate on Structural Phase Transition
in a Conducting Polymer during Ion Injection and Water Intake: A View
from a Computational Microscope. ACS Appl. Electron.
Mater..

[14] Siemons N., Pearce D., Yu H., Tuladhar S. M., LeCroy G. S., Sheelamanthula R., Hallani R. K., Salleo A., McCulloch I., Giovannitti A. (2023). Controlling Swelling in Mixed Transport Polymers
through Alkyl Side-Chain Physical Cross-Linking. Proc. Natl. Acad. Sci. U. S. A..

[15] Moser M., Hidalgo T. C., Surgailis J., Gladisch J., Ghosh S., Sheelamanthula R., Thiburce Q., Giovannitti A., Salleo A., Gasparini N. (2020). Side Chain Redistribution
as a Strategy to Boost Organic Electrochemical Transistor Performance
and Stability. Adv. Mater..

[16] Moser M., Gladisch J., Ghosh S., Hidalgo T. C., Ponder J. F., Sheelamanthula R., Thiburce Q., Gasparini N., Wadsworth A., Salleo A. (2021). Controlling Electrochemically
Induced Volume Changes in Conjugated Polymers by Chemical Design:
From Theory to Devices. Adv. Funct. Mater..

[17] Moser M., Savagian L. R., Savva A., Matta M., Ponder J. F., Hidalgo T. C., Ohayon D., Hallani R., Reisjalali M., Troisi A., Wadsworth A., Reynolds J. R., Inal S., McCulloch I. (2020). Ethylene Glycol-Based
Side Chain Length Engineering in Polythiophenes and Its Impact on
Organic Electrochemical Transistor Performance. Chem. Mater..

[18] Onorato J. W., Wang Z., Sun Y., Nowak C., Flagg L. Q., Li R., Dong B. X., Richter L. J., Escobedo F. A., Nealey P. F., Patel S. N., Luscombe C. K. (2021). Side Chain
Engineering Control of
Mixed Conduction in Oligoethylene Glycol-Substituted Polythiophenes. J. Mater. Chem. A.

[19] Matta M., Wu R., Paulsen B. D., Petty A. J., Sheelamanthula R., McCulloch I., Schatz G. C., Rivnay J. (2020). Ion Coordination and
Chelation in a Glycolated Polymer Semiconductor: Molecular Dynamics
and X-Ray Fluorescence Study. Chem. Mater..

[20] Elliott J. D., Troisi A., Carbone P. (2020). A QM/Md Coupling Method to Model
the Ion-Induced Polarization of Graphene. J.
Chem. Theory Comput..

[21] Wei Z., Elliott J. D., Papaderakis A. A., Dryfe R. A. W., Carbone P. (2024). Relation between
Double Layer Structure, Capacitance, and Surface Tension in Electrowetting
of Graphene and Aqueous Electrolytes. J. Am.
Chem. Soc..

[22] Elliott J. D., Chiricotto M., Troisi A., Carbone P. (2023). Do Specific Ion Effects
Influence the Physical Chemistry of Aqueous Graphene-Based Supercapacitors?
Perspectives from Multiscale QMMD Simulations. Carbon.

[23] Di
Pasquale N., Finney A. R., Elliott J. D., Carbone P., Salvalaglio M. (2023). Constant Chemical Potential–Quantum Mechanical–Molecular
Dynamics Simulations of the Graphene–Electrolyte Double Layer. J. Chem. Phys..

[24] Papaderakis A. A., Leketas M., Wei Z., Hwang I., Carbone P., Juel A., Dryfe R. A. W. (2024). Electrowetting
of Carbon-Based Materials
for Advanced Electrochemical Technologies. ChemElectroChem.

[25] Elliott J. D., Papaderakis A. A., Dryfe R. A. W., Carbone P. (2022). The Electrochemical
Double Layer at the Graphene/Aqueous Electrolyte Interface: What We
Can Learn from Simulations, Experiments, and Theory. J. Mater. Chem. C.

[26] Burke C., Landi A., Troisi A. (2024). The Dynamic
Nature of Electrostatic
Disorder in Organic Mixed Ionic and Electronic Conductors. Mater. Horiz..

[27] Landi A., Reisjalali M., Elliott J. D., Matta M., Carbone P., Troisi A. (2023). Simulation
of Polymeric Mixed Ionic and Electronic
Conductors with a Combined Classical and Quantum Mechanical Model. J. Mater. Chem. C.

[28] Jeanmairet G., Rotenberg B., Salanne M. (2022). Microscopic Simulations of Electrochemical
Double-Layer Capacitors. Chem. Rev..

[29] Melander M. M., Wu T., Weckman T., Honkala K. (2024). Constant Inner Potential DFT for
Modelling Electrochemical Systems under Constant Potential and Bias. Npj Comput. Mater..

[30] Chai Z., Si R., Chen M., Teobaldi G., O’Regan D. D., Liu L.-M. (2024). Minimum Tracking
Linear Response Hubbard and Hund Corrected
Density Functional Theory in CP2K. J. Chem.
Theory Comput..

[31] Bi S., Banda H., Chen M., Niu L., Chen M., Wu T., Wang J., Wang R., Feng J., Chen T., Dinca M., Kornyshev A. A., Feng G. (2020). Molecular Understanding
of Charge Storage and Charging Dynamics in Supercapacitors with MOF
Electrodes and Ionic Liquid Electrolytes. Nat.
Mater..

[32] Zeng L., Wu T., Ye T., Mo T., Qiao R., Feng G. (2021). Modeling Galvanostatic
Charge–Discharge of Nanoporous Supercapacitors. Nat. Comput. Sci..

[33] Scalfi L., Dufils T., Reeves K. G., Rotenberg B., Salanne M. (2020). A Semiclassical Thomas–Fermi
Model to Tune the
Metallicity of Electrodes in Molecular Simulations. J. Chem. Phys..

[34] Yang X.-H., Zhuang L., Cheng J. (2025). Decoding the
Influence of Monomer
Structures on the Electrical Double Layer of Alkaline Fuel Cells. Chem. Sci..

[35] Tavernelli I., Vuilleumier R., Sprik M. (2002). Ab Initio Molecular Dynamics for
Molecules with Variable Numbers of Electrons. Phys. Rev. Lett..

[36] Bonnet N., Morishita T., Sugino O., Otani M. (2012). First-Principles
Molecular
Dynamics at a Constant Electrode Potential. Phys. Rev. Lett..

[37] Hu X., Chen S., Chen L., Tian Y., Yao S., Lu Z., Zhang X., Zhou Z. (2022). What Is the Real Origin of the Activity
of Fe–N–C Electrocatalysts in the O2 Reduction Reaction?
Critical Roles of Coordinating Pyrrolic N and Axially Adsorbing Species. J. Am. Chem. Soc..

[38] Zhao Q., Martirez J. M. P., Carter E. A. (2021). Revisiting
Understanding of Electrochemical
CO2 Reduction on Cu (111): Competing Proton-Coupled Electron Transfer
Reaction Mechanisms Revealed by Embedded Correlated Wavefunction Theory. J. Am. Chem. Soc..

[39] Bai X., Zhao X., Zhang Y., Ling C., Zhou Y., Wang J., Liu Y. (2022). Dynamic Stability
of Copper Single-Atom
Catalysts under Working Conditions. J. Am. Chem.
Soc..

[40] Xia Z., Xiao H. (2023). Grand Canonical Ensemble Modeling of Electrochemical Interfaces Made
Simple. J. Chem. Theory Comput..

[41] Melander M. M., Kuisma M. J., Christensen T. E. K., Honkala K. (2019). Grand-Canonical Approach
to Density Functional Theory of Electrocatalytic Systems: Thermodynamics
of Solid-Liquid Interfaces at Constant Ion and Electrode Potentials. J. Chem. Phys..

[42] Makki H., Burke C. A., Troisi A. (2023). Microstructural
Model of Indacenodithiophene-Co-Benzothiadiazole
Polymer: Π-Crossing Interactions and Their Potential Impact
on Charge Transport. J. Phys. Chem. Lett..

[43] Eslami H., Müller-Plathe F. (2007). Molecular
Dynamics Simulation in the Grand Canonical
Ensemble. J. Comput. Chem..

[44] Papadopoulou A., Becker E. D., Lupkowski M., van Swol F. (1993). Molecular Dynamics
and Monte Carlo Simulations in the Grand Canonical Ensemble: Local
versus Global Control. J. Chem. Phys..

[45] Lupkowski M., van Swol F. (1991). Ultrathin
Films under Shear. J. Chem. Phys..

[46] Elstner M., Porezag D., Jungnickel G., Elsner J., Haugk M., Frauenheim T., Suhai S., Seifert G. (1998). Self-Consistent-Charge
Density-Functional Tight-Binding Method for Simulations of Complex
Materials Properties. Phys. Rev. B.

[47] Koskinen P., Mäkinen V. (2009). Density-Functional
Tight-Binding for Beginners. Comput. Mater.
Sci..

[48] Sunny S., Shah S., Garg M., Zozoulenko I., Ghosh S. (2024). Microscopic Insights of Electrochemical
Switching of Poly (Benzimidazobenzophenanthroline)­(Bbl)
Thin Film: A Molecular Dynamics Study. Macromolecules.

[49] Makki H., Troisi A. (2022). Morphology of Conducting Polymer Blends at the Interface
of Conducting and Insulating Phases: Insight from PEDOT: PSS Atomistic
Simulations. J. Mater. Chem. C.

[50] Berendsen H. J. C., van der Spoel D., van Drunen R. (1995). GROMACS: A
Message-Passing Parallel Molecular Dynamics Implementation. Comput. Phys. Commun..

[51] Hoover W. G. (1985). Canonical
Dynamics: Equilibrium Phase-Space Distributions. Phys. Rev. A.

[52] Parrinello M., Rahman A. (1981). Polymorphic Transitions in Single Crystals: A New Molecular
Dynamics Method. J. Appl. Phys..

[53] Verlet L. (1967). Computer“Experiments”
on Classical Fluids. I. Thermodynamical Properties of Lennard-Jones
Molecules. Phys. Rev..

[54] Darden T., York D., Pedersen L. (1993). Particle Mesh
Ewald: An N Log (N)
Method for Ewald Sums in Large Systems. J. Chem.
Phys..

[55] Berendsen H. J. C., Grigera J. R., Straatsma T. P. (1987). The Missing
Term in Effective Pair
Potentials. J. Phys. Chem..

[56] Jorgensen W. L., Maxwell D. S., Tirado-Rives J. (1996). Development
and Testing of the OPLS
All-Atom Force Field on Conformational Energetics and Properties of
Organic Liquids. J. Am. Chem. Soc..

[57] Aradi B., Hourahine B., Frauenheim T. (2007). DFTB+, a Sparse Matrix-Based Implementation
of the DFTB Method. J. Phys. Chem. A.

