# Supplementary material for: Simulation of Charge Distribution and Microstructure in Semicrystalline Polymeric Ionic-Electronic Conductors Using Classical Simulation at Constant Electrochemical Potential
Source: J Chem Theory Comput. 2026 Mar 6;22(6):3061–71. doi: 10.1021/acs.jctc.5c02111 (PMC13019674; doi:10.1021/acs.jctc.5c02111)
Supplement: Supplementary file 1 [file ct5c02111_si_001.pdf]

Simulation of charge distribution and microstructure in semicrystalline polymeric ionic-electronic conductors using classical simulation at constant electrochemical potential

Zixuan Wei[a], Hesam Makki[a,b], Paola Carbone[c], Alessandro Troisi[a]*

[a] Department of Chemistry, University of Liverpool, Crown Street, L69 7ZD, Liverpool (UK)

[b]Department of Chemical Engineering, University of Bath, BA2 7AY, Bath (UK)

[c]Department of Chemistry, The University of Manchester, Oxford Road, M13 9PL, Manchester (UK)

Supporting Information

∗To whom correspondence should be addressed. Email: zixuan.wei@liverpool.ac.uk

Contents

List of Figures

Figure S1. Correlation of the absolute value of reduced chain HOMO energy with chain ionisation energy (E(A^+)-E(A)) for ca. 300 samples……………………………………….………..…..…………..S3

Figure S2. (a) The RDFs between polymer and Cl^- with the case of standard SPC/E (the blue line) and with the case of SPC/E with the excess charge on oxygen (the orange line). (b) The RDFs between polymer and Na^+ with the case of standard SPC/E (the blue line) and with the case of SPC/E with the excess charge on oxygen (the orange line)……………………………………….………………………S4

Figure S3. The simulation temperature (with and without GC moves) as the function of time..………S5

Figure S4. The varied chain numbers under different $\Delta\Phi$ as the function of time……………………S6

Figure S5. The distribution of the $\theta_{\pi-\pi}$ with the $D_{shortest}^{\pi-\pi}$ in thiophene rings under different oxidation levels of 31%, 36%, 49%, 53%, 63% and 100% (heatmap), while the top plot of each figure is the distribution of $D_{shortest}^{\pi-\pi}$ and the right plot of each figure is the distribution of $\theta_{\pi-\pi}$…………………S7

Figure S6. The distribution of the $\theta_{lamellae}$ with the $D_{shortest}^{lamellae}$ between lamellae under different oxidation levels of 31%, 36%, 49%, 53%, 63% and 100% (heatmap), while the top plot of each figure is the distribution of $D_{shortest}^{lamellae}$ and the right plot of each figure is the distribution of $\theta_{lamellae}$………S8

Figure S7. (a-d) The data of the three figures in each column are under the same $\Delta\Phi$ as shown at the top of this column. The first row of figures shows the charge distribution of each chain as a function of time, where the dark blue and white represent the oxidized and reduced states, respectively. The second row of figures represents the time-average charge of each chain as a function of the $\Delta\Phi$. The dashed purple line is the overall time-average charge value in all chains. The dark blue band represents the standard deviation. The third row of figures represents the time-average HOMO energy of each chain as a function of the $\Delta\Phi$. The light blue band represents the standard deviation………….……………….S9

S1. A validation test of the approximation of using HOMO energy

We selected a case at 49% oxidation level and sampled randomly chosen polymer chain A at different timesteps. For each selected chain, we computed both the HOMO energy and the corresponding $E(A^{+})-E(A)$ using the same geometry and embedding environment. Although the number of samples is moderate (300 samples), the results exhibit a strong correlation between the two quantities, as shown in **Figure S1**.

This strong correlation supports the validity of using the HOMO energy as a proxy for the $E(A^{+})-E(A)$ for polymer chains of the length and under the environmental conditions considered in this study.

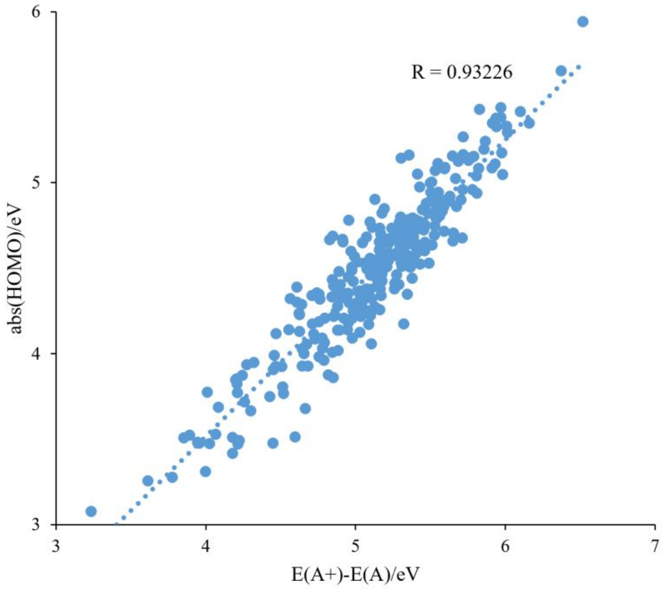

Figure S1. Correlation of the absolute value of reduced chain HOMO energy with chain ionisation energy ($E(A^{+})$-E(A)) for ca. 300 samples.

S2. A validation test of neutralized approach on the water oxygen

To explicitly assess the validity of this neutralized approach (mean-field approach), we performed a direct comparison at the highest doping level considered in this work (100% oxidation level). We contrast two otherwise identical systems: (i) charge neutralization via a uniform background charge on SPC/E water oxygen atoms, and (ii) charge neutralization via additional explicit counter-ions (10 extra Cl^- ions) using standard SPC/E water. After equilibration, we analyzed the last 5 ns of NPT trajectories and computed the radial distribution functions (RDFs) between the polymer and Na^+/Cl^- ions.

As shown in Figure S2 below, the RDFs obtained from the two neutralization schemes are nearly indistinguishable, indicating that the mean-field background charge does not significantly alter local ion–polymer correlations or ion-pairing behavior at the doping levels explored in this study. This comparison demonstrates that the uniform background charge approximation does not artificially damp local structural reorganization in the low doping levels relevant here.

We emphasize that for high doping levels, where strong counter-ion condensation and localized screening dominate the electrostatics, the mean-field approach would no longer be sufficient. Such doping levels require explicit treatment of counter-ion migration and are beyond the scope of the present work.

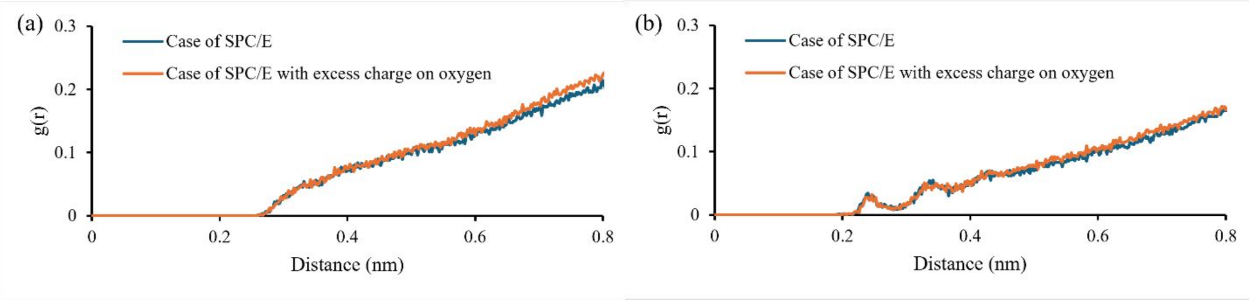

Figure S2. (a) The RDFs between polymer and Cl^- with the case of standard SPC/E (the blue line) and with the case of SPC/E with the excess charge on oxygen (the orange line). (b) The RDFs between polymer and Na^+ with the case of standard SPC/E (the blue line) and with the case of SPC/E with the excess charge on oxygen (the orange line).

S3. A validation test of temperature

To assess the stability of GC-MD and exclude artificial heating effects arising from force discontinuities, we checked the system temperature throughout the GC-MD simulation. **Figure S3** here shows the temperature as a function of time for representative cases at 0% oxidation level (without redox reactions) and 49% oxidation level (with frequent redox fluctuations). The temperature of two cases remains stable and fluctuates narrowly around the target value of 298.15 K, indicating that the temperature does not significantly affect by the non-conservative force jump and that the simulation remains stable.

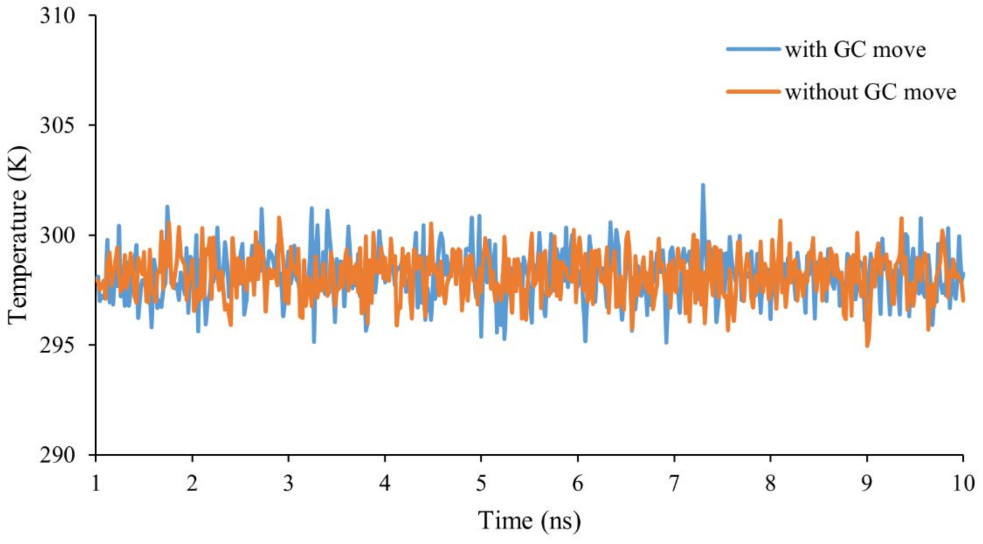

Figure S3. The simulation temperature (with and without GC moves) as the function of time.

S4. varied chain numbers

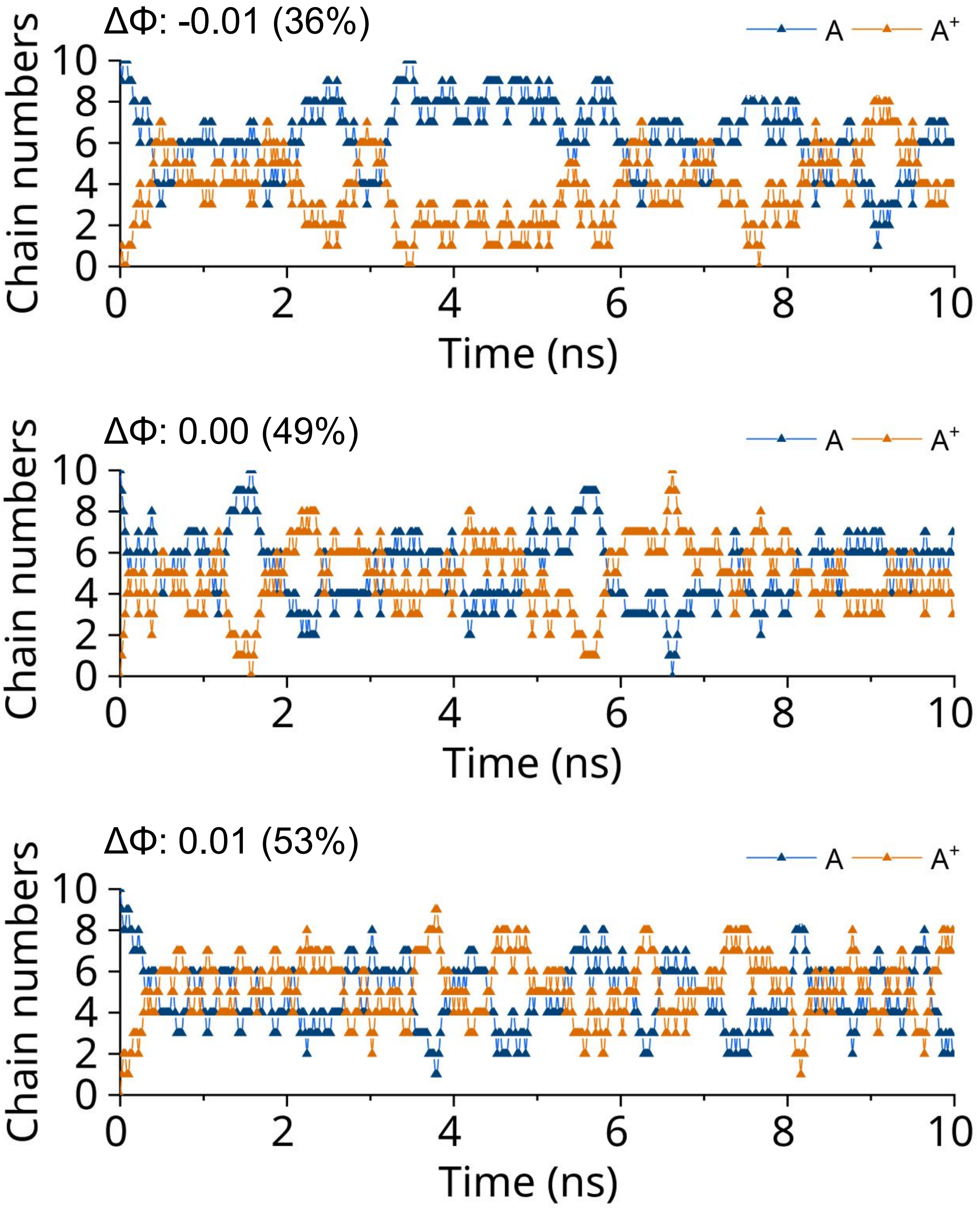

Figure S4. The varied chain numbers under different ΔΦ as the function of time.

S5. The distribution of the $\theta_{\pi-\pi}$ with the $D_{shortest}^{\pi-\pi}$

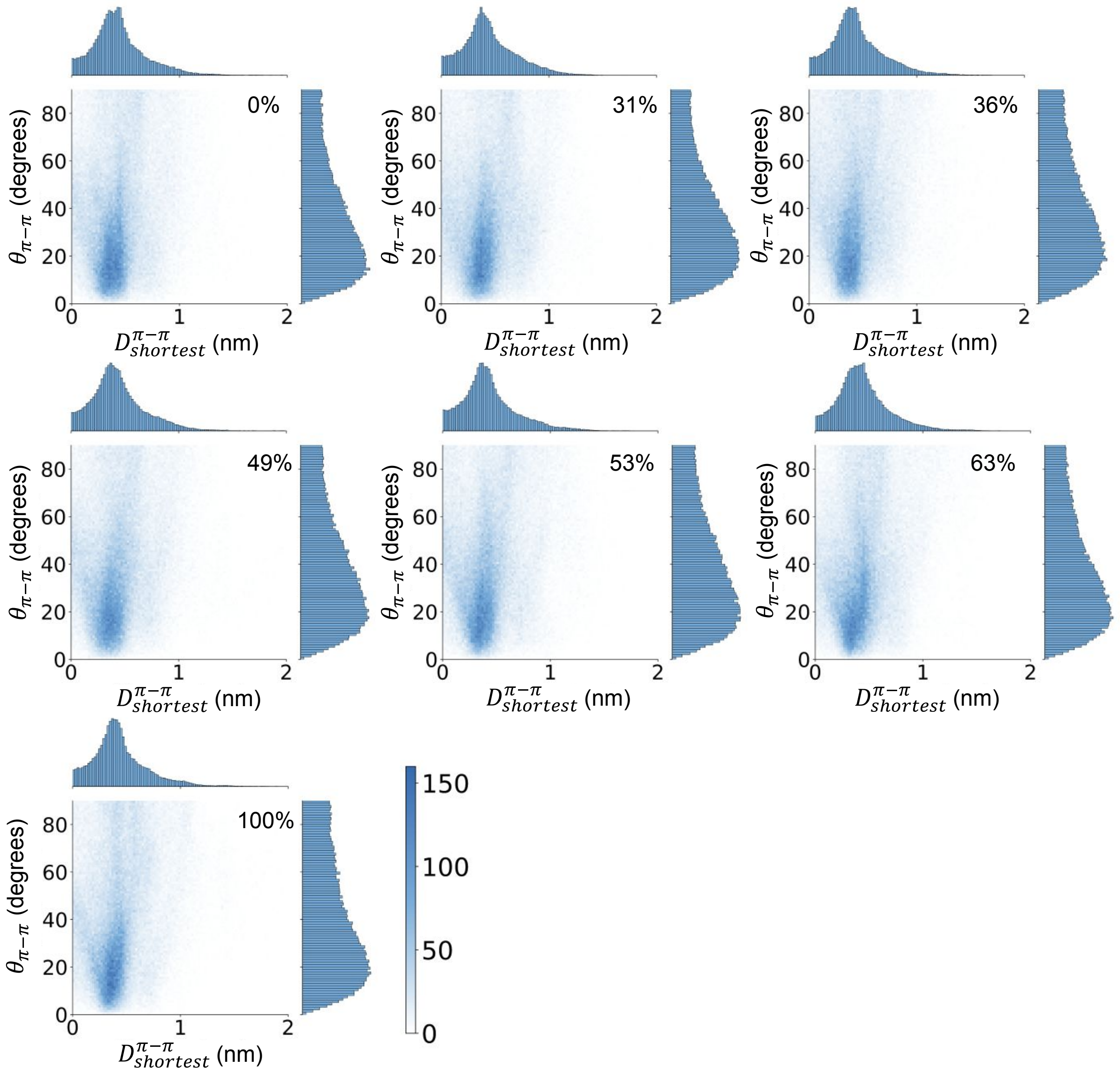

Figure S5. The distribution of the $\theta_{\pi-\pi}$ with the $D_{shortest}^{\pi-\pi}$ in thiophene rings under different oxidation levels of 31%, 36%, 49%, 53%, 63% and 100% (heatmap), while the top plot of each figure is the distribution of $D_{shortest}^{\pi-\pi}$ and the right plot of each figure is the distribution of $\theta_{\pi-\pi}$.

S6. The distribution of the $\theta_{lamellae}$ with the $D_{shortest}^{lamellae}$

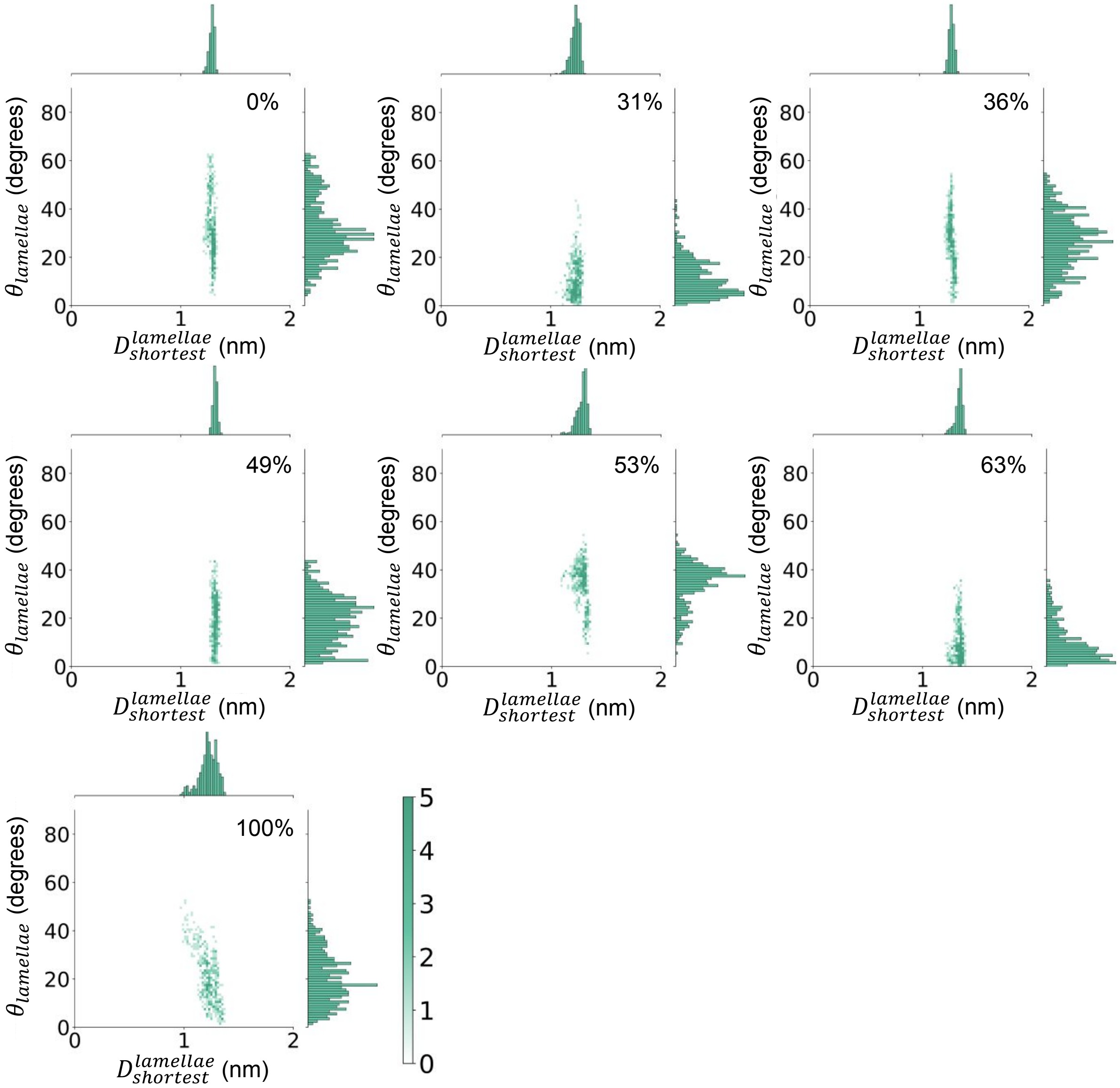

Figure S6. The distribution of the $\theta_{lamellae}$ with the $D_{shortest}^{lamellae}$ between lamellae under different oxidation levels of 31%, 36%, 49%, 53%, 63% and 100% (heatmap), while the top plot of each figure is the distribution of $D_{shortest}^{lamellae}$ and the right plot of each figure is the distribution of $\theta_{lamellae}$.

S7. Charge distribution in varied $\Delta\Phi$

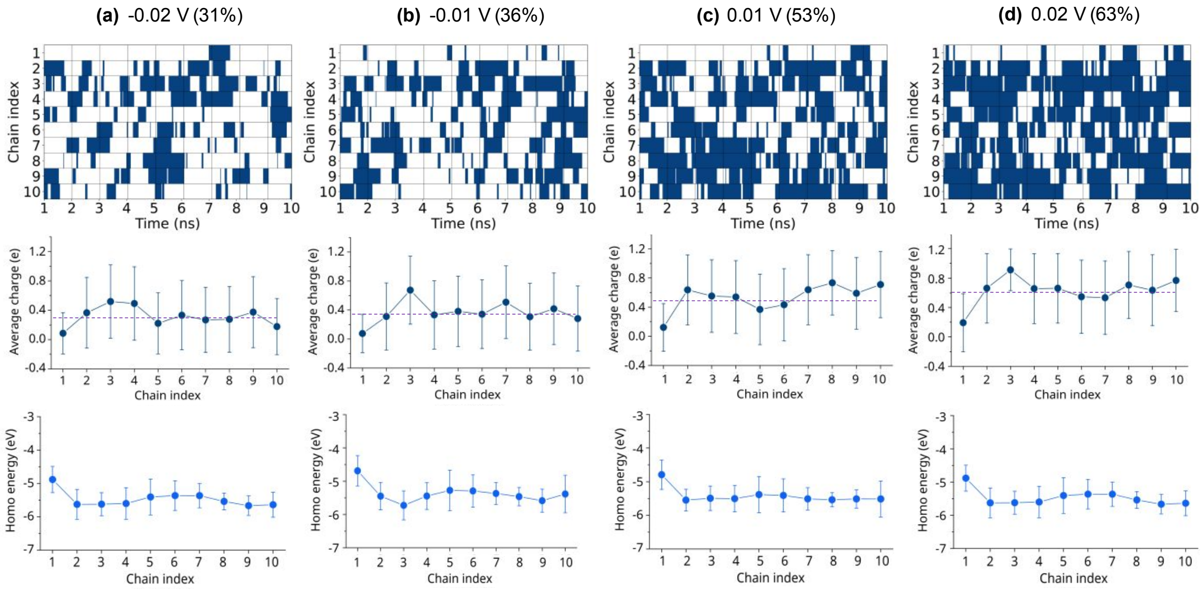

Figure S7. (a-d) The data of the three figures in each column are under the same $\Delta\Phi$ as shown at the top of this column. The first row of figures shows the charge distribution of each chain as a function of time, where the dark blue and white represent the oxidized and reduced states, respectively. The second row of figures represents the time-average charge of each chain as a function of the $\Delta\Phi$ with the standard deviation. The dashed purple line is the overall time-average charge value in all chains. The third row of figures represents the time-average HOMO energy of each chain as a function of the $\Delta\Phi$ with the standard deviation.
